# Pain at the Slaughterhouse in Ruminants with a Focus on the Neurobiology of Sensitisation

**DOI:** 10.3390/ani11041085

**Published:** 2021-04-10

**Authors:** Daniel Mota-Rojas, Fabio Napolitano, Ana Strappini, Agustín Orihuela, Marcelo Daniel Ghezzi, Ismael Hernández-Ávalos, Patricia Mora-Medina, Alexandra L. Whittaker

**Affiliations:** 1 Neurophysiology, Behavior and Animal Welfare Assessment, DPAA, Universidad Autónoma Metropolitana, Xochimilco Campus, 04960 Ciudad de México, Mexico; 2 Scuola di Scienze Agrarie, Forestali, Alimentari ed Ambientali, Università degli Studi della Basilicata, 85100 Potenza, Italy; fabio.napolitano@unibas.it; 3 Animal Science Institute, Faculty of Veterinary Sciences, Universidad Austral de Chile, 5090000 Valdivia, Chile; anastrappini@uach.cl; 4 Facultad de Ciencias Agropecuarias, Universidad Autónoma del Estado de Morelos, 62209 Cuernavaca, Mexico; aorihuela@uaem.mx; 5 Animal Welfare Area, Faculty of Veterinary Sciences (FCV), Universidad Nacional del Centro de la Provincia de Buenos Aires (UNCPBA), 7000 Buenos Aires, Argentina; ghezzi@vet.unicen.edu.ar; 6 Facultad de Estudios Superiores Cuautitlán, Universidad Nacional Autónoma de México (UNAM), 54714 Cuautitlan Izcalli, Mexico; mvziha@hotmail.com (I.H.-Á.); mormed2001@yahoo.com.mx (P.M.-M.); 7 School of Animal and Veterinary Sciences, University of Adelaide, Roseworthy, SA 5116, Australia

**Keywords:** pain, abattoir, sensitisation, stunning, cattle, river buffalo, animal welfare, Halal, Shechita, Kosher

## Abstract

**Simple Summary:**

We pose based on a fundamental science examination that events that occur around the time of slaughter have the potential to intensify the pain response, through a process called sensitisation, or an exaggerated response to painful stimuli. Health conditions which result in inflammation, injuries arising from transport and handling and exaggerated fear responses may all be present at the slaughterhouse. Whilst there is limited evidence of a direct effect of these on the processes of sensitisation in animals at slaughter, by analogy with the human neurobiology literature the connection seems plausible. In this review we outline the biology of such a response, and the rationale for suggestion of a possible linkage between events at slaughter and a heightened animal pain response.

**Abstract:**

We pose, based on a neurobiological examination, that events that occur around the time of slaughter have the potential to intensify the pain response, through the processes of sensitisation and enhanced transmission. Sensitisation, or an enhanced response to painful stimuli, is a well-discussed phenomenon in the human medical literature, which can arise from previous injury to an area, inflammatory reactions, or previous overstimulation of the stress axes. A number of events that occur prior to arrival at, or in the slaughterhouse, may lead to presence of these factors. This includes previous on-farm pathology, injuries arising from transport and handling and lack of habituation to humans. Whilst there is limited evidence of a direct effect of these on the processes of sensitisation in animals at slaughter, by analogy with the human neurobiology literature the connection seems plausible. In this review a neurobiological approach is taken to discuss this hypothesis in the light of basic science, and extrapolations from existing literature on the slaughter of ruminants. To confirm the postulated link between events at slaughter, and processes of hypersensitisation, further dedicated study is required.

## 1. Introduction

Recently the International Association for the Study of Pain (IASP) revised the definition of pain to “an unpleasant sensory and emotional experience associated with or resembling that associated with, actual or potential tissue damage” [1]. Pain experienced by animals at abattoirs has long been a topic of discussion and may arise at multiple stages of the slaughter process through transport, lairaging as well as slaughter itself. 

In ruminants the common method of causing death is through sticking. This involves the cutting of the soft tissues of the neck, carotid arteries and jugular veins for bleeding. It is imperative that all major blood vessels are severed to ensure time to death is reduced. The sticking process causes significant nociceptor stimulation [2], in addition to causing distress through method of restraint, as well as through aspiration of blood [3,4]. 

Other factors associated with pain prior to death include the use of electric drivers or prods, hot-iron branding and trauma caused by sticks or tubes that can inflict painful lesions and produce over-stimulation of nociceptors [5,6,7]. These events can also result in central and peripheral sensitisation that causes animals to perceive pain more intensely [5,6,7].

Antemortem stunning is designed to minimise pain and distress by rendering animals insensitive prior to sticking. Stunning techniques prevent correct functioning of neurons in the brain leading to a state of unconsciousness which may be reversible or irreversible [8]. However, whilst the purpose of stunning is to reduce welfare impact, stunning methods have also been a source of controversy with regard to impact on welfare [9]. Mechanical methods like penetrating captive bolt guns, are prone to technical problems that can affect the quality of stunning [10], for example air pressure in pneumatic stunning devices must be sufficient to propel the bolt at an appropriate velocity to cause enough damage to the brain to cause insensibility.

Therefore, the aim of this article is to review the neurobiology of antemortem pain in ruminants, the procedures that cause pain during pre-slaughter handling and those that can mitigate it, and the consequences for the welfare of the animal. A particular focus of the review is on how events occurring at slaughter could impact on hyperalgesia or sensitisation, and the consequences of this for the welfare of the animal. The concerns of an inadequate desensitisation and slaughter without stunning are also discussed.

## 2. Consciousness

A key consideration in relation to pain at slaughter is the point at which consciousness is lost since this loss prevents the animal experiencing pain and fear [8]. Two events cause this unconsciousness: stunning and/or hypovolemic shock caused by blood loss from sticking.

Consciousness is generally considered to consist of two components; the content, represented by awareness of the environment and oneself, and the level (wakefulness) [11]. The former requires functioning of the cerebral cortex and connecting networks to subcortical structures such as the thalamus [11]. In the context of slaughter, perception of the environment is particularly relevant since information from the senses may all contribute to a fear response, for example smell of blood or fear pheromones, stress vocalisations from other animals and visual threats. Due to the lack of specific studies any relationship between these factors and any effect on stunning efficiency is only speculative. The wakefulness component depends on the ascending reticular system in the upper brainstem tegmentum and thalamus [11]. Projections in this system travel to the cortex and enable cortical functioning. Therefore, lesions to large areas of the cerebral cortex, the reticular formation itself or the ascending projections, created during stunning or slaughter lead to unconsciousness [8]. As a consequence, it is these brain regions that are targeted by various stunning methods [12,13].

The processes of nociception described below are ’pre-conscious’. However, pain perception or the emotional experience of pain is described as a ‘conscious sensation’ since it requires a functioning cerebral cortex [14]. Therefore, it is generally assumed that an unconscious state implies that no pain is ‘felt’ [8]. There is however some contrary evidence from human studies suggesting that a low level of residual pain perception may exist in an unconscious state [15].

## 3. The Neurobiology of Pain 

**Figure 1:**
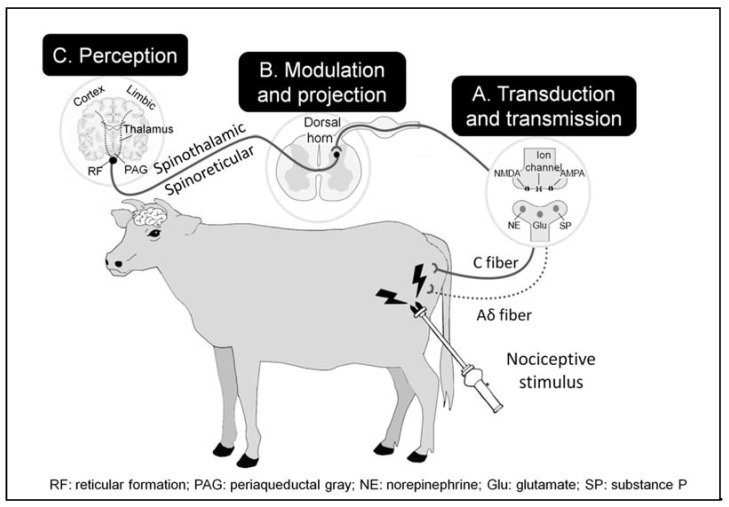
Schematic representation of nociceptor stimulation in response to a harmful stimulus produced by an electric drive. The first stage of transduction occurs when the harmful stimulus created by the electric drive activates nociceptors and is converted to an electrical impulse. The second stage is transmission, where the information is channelled through two primary efferent nociceptive neurons; the C-fibres (or C-polymodal nociceptors) and A-delta fibres [18]. Attenuation of the response occurs in the dorsal horn of the spinal cord and is called modulation. Finally, processing and integration of the response occurs so pain is perceived. N-Methyl-D-aspartate receptor (NMDA), and α-amino-3-hydroxy-5-methyl-4-isoxazolepropionic acid (AMPA).

The nociceptive pathways and neurophysiological mechanisms associated with pain are similar across mammalian species since the neuronal processes involved in the stages of conduction, modulation and integration develop through a chain of neurons with three pathways [16,17,18]. The following provides a brief overview of the important features of the pain pathway and its modulation as it relates to events at slaughter. Figure 1 summarises the pain pathway described in the following sections.

### 3.1. Transduction and Transmission

Transduction is the first element in the pain pathway. This refers to the transformation of harmful stimuli, such as those of thermal, chemical, or mechanical origin into electrical impulses by nociceptors [6]. Nociceptors are sensory neurons found in the skin, muscles, bones and viscera [5]. During sticking or handling the animal with sharp tools or electrical devices (goading), tissue damage occurs creating a harmful stimulus and consequent nociceptor activation. Opening of ionic Ca^2+^, K^+^ or Na^2+^ channels follow to produce electrical impulses that transmit the nociceptive signal along the neuronal axons to the spinal cord, brainstem, thalamus and cortex [19]. 

Nociceptors can be differentiated based on their expression of a series of channels that confer a type of sensitivity to various stimuli, such as heat or cold, and acid or chemical irritants. The channels most often observed are transient receptor potential vanilloid channel (TRPV1), transient receptor potential melastatin (TRPM8) and TRPA1. These distinct classes of nociceptors are associated with specific functions and stimuli, although variation in pain intensity may also depend on the tissues and nerve fibers involved. The nociceptors activated at this stage are called mechanoreceptors or mechanonociceptors that are activated by touch and pressure. They consist primarily of myelinated Aδ or type C unmyelinated fibers [5]. Usually silenced, once nociceptors reach their threshold of activation, they will respond in proportion to the pressure applied. 

Transmission is the next stage in pain signalling. This occurs when the electrical signal generated by the nociceptors is conducted along the axons of first-order neurons towards synapses with second-order neurons. Second-order neurons form synapses in the dorsal horn of the spinal cord using the so-called “Lissauer tract”, which consists mainly of a series of propriospinal fibres in which Aδ and C fibres predominate. The Aδ nerve fibres described as non-nociceptive participate in nociceptive phenomena when there is sensitisation (see later) [20]. 

Transmission of the nervous impulse occurs along high-velocity, myelinated conduction fibres (12–30 m/sec) which are associated with “first pain”, also described as throbbing pain. The signals from the silent, polymodal nociceptors transported by demyelinated, slow conductivity C fibres (0.5–2 m/sec) are responsible for “second pain”, which is characterised as penetrating, visceral pain that has been described as “burning”. A third class of Aβ nerve fibres, such as those of touch (50 m/sec), may be activated at low stimulus thresholds, but contribute to transmitting pain signals only in cases of peripheral sensitisation [20].

### 3.2. Hyperalgesia

**Figure 2:**
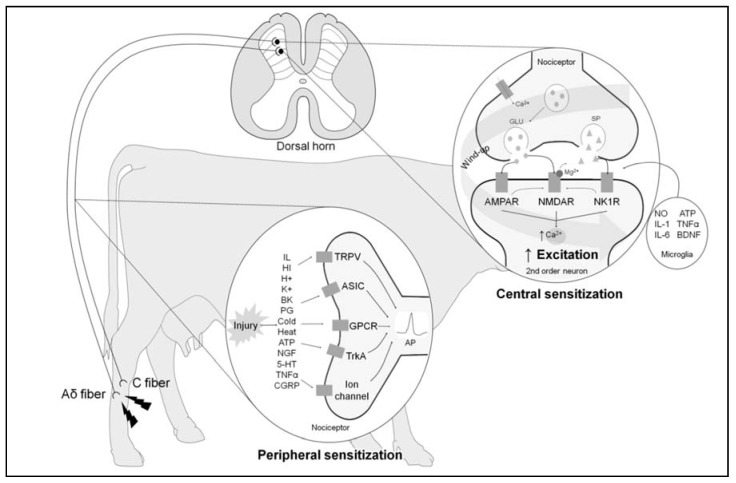
Peripheral and central sensitisation processes. After tissue injury, multiple chemical mediators (e.g., interleukin (IL), histamine (HI), H^+^, K^+^, bradykinin (BK), prostaglandin (PG), cold, heat, adenosine triphosphate (ATP), nerve growth factor (NGF), serotonin (5-HT), tumour necrosis factor (TNFα) and calcitonin gen related peptide (CGRP)), known as “sensitizing soup”, activate receptors in nociceptors (transient receptor potential cation channel (TRPV), acid-sensing ion channels (ASIC), G-protein-coupled receptors (GPCR), tropomyosin receptor kinase A (TrkA) and ion channel), increasing the ratio of action potentials that results in continuous depolarisation and increased sensitisation of peripheral pain receptors. When harmful stimuli reach second-order neurons in the dorsal horn of the spinal cord, excitatory substances like glutamate (GLU) and substance P (SP) are released by the nociceptors into secondary neurons. Activation of α-amino-3-hydroxy-5-methyl-4-isoxazolepropionic acid receptor (AMPAR), N-Methyl-D-aspartate receptor (NMDAR), neurokinin-1 receptor (NK1R) and simultaneous changes increase intracellular Ca^2+^ concentrations, enhancing transmission and excitation, together with the so-called wind-up phenomenon after repeated activation of nociceptive fibres. Other mediators produced by glial cells (e.g., nitric oxide (NO), IL-1, IL-6, ATP, TNFα and brain-derived neurotrophic factor (BDNF)) contribute to the central sensitisation and amplification of responsiveness to painful sensations and the development of hyperalgesia to sensory inputs.

Since nociceptors are heterogeneous and respond to multiple types of stimuli, it is possible for a stimulus acting via one modality, such as pressure, to alter the response of the nociceptor to other modalities, such as temperature [21]. Furthermore, tissue injury can cause an increased sensitivity of the afferent nerves to stimulation, so-called peripheral sensitisation. This “primary hyperalgesia” results from the production of neuropeptides by the nociceptors and upregulation of existing local receptors rendering them more responsive. In contrast, central sensitisation may occur in tissue adjacent to the injured area. Increased responsiveness in this area implies that this hypersensitivity arises due to a central mechanism acting via the dorsal horn and this is termed secondary hyperalgesia [21]. Both primary and secondary hyperalgesia (Figure 2) are key elements of concern during slaughter due to the potential for a heightened sensitivity, or exaggerated response to pain, arising as a result of events occurring at slaughter.

Released glutamate bonds to α-amino-3-hydroxy-5-methyl-4-isoxazolepropionic acid (AMPA) receptors, generating post-synaptic excitatory potentials involving C fibre peripheral nociceptors that repetitively stimulate neurons in the dorsal horn. This results in mass depolarisation of neurons, the consequence of which is the release of neurotransmitters like substance P and brain-derived neurotrophic factor. These substances produce slow excitatory potentials. When substance P is released by high-threshold fibres, CGRP is simultaneously released and acts to extend the zone of the spinal cord capable of releasing CGRP. This phenomenon contributes to the excitability and activation of glutamate receptors like NMDA [22]. The activation of these receptors eliminates Mg^2+^, increasing intracellular Ca^2+^ and promoting production of prostanoids and nitrous oxide. This is accompanied by activation of wide dynamic range neurons (WDR) and hyper-excitability of the specific nociceptive neurons (NNSO). Excitation thresholds are lowered and two events may occur: (1) an exaggerated response to painful stimuli (hyperalgesia) or (2) pain perception after exposure to stimuli that are not normally painful (allodynia) [22].

Lesions or trauma also stimulate a neuroendocrine reaction due to the intricate connections between the two systems. This reaction has three components: (1) the sympathetic-adrenomedullary (SAM) response mediated by catecholamines such as dopamine, noradrenaline and adrenaline, (2) Corticotropin-releasing hormone (CRH) activation of the hypothalamic-pituitary-adrenal (HPA) axis leading to production of glucocorticoids and (3) activation of the locus coeruleus and the noradrenergic limbic brain [2,23,24]. Limbic structures, including the hippocampus, amygdala and pre-frontal cortex have an inhibitory influence by resetting the HPA axis following a stressful event [25]. Activation of these systems does not directly initiate pain signalling but has an indirect influence via effects on neuroimmune cells. A key action is via mast cells which become activated and infiltrate peripheral injured tissues in response to CRH. This leads to enhanced peripheral nociceptor interaction, increased stimulation or ‘priming’, with subsequent increased ascendant pain signalling and reduced descendant inhibition. Sensitisation occurs as a result [25]. 

Tissue injury also leads to chemical release from non-neuronal cells, such as neutrophils, and from sensory terminals of primary afferent fibres. Mediators such as histamine, bradykinin, acetylcholine, serotonin and substance P are generated as part of this inflammatory process. Whilst these chemicals may not activate nociceptors directly, they are thought to enhance pain sensation by increasing rate of action potential firing and reducing the firing threshold in the nociceptive neurons [21,26]. Frequency of firing corresponds to pain intensity [27], hence increased firing enhances pain and mediates sensitisation. Meanwhile, serotonin released by mastocytes and platelets participates in the first phases of nociception and acute inflammation. This release, accompanied by activation of the sympathetic nervous system, triggers the release of noradrenaline that accelerates sensitisation of the nociceptors and the subsequent release of the CGRP-related peptide and substance P. These peptides generate degranulation of mastocytes, vasodilatation and oedema in tissues around the damaged area [22]. These events in turn lead to sensitisation of nearby sensory nerve endings as described previously [25]. It is therefore important to control these substances in order to reduce pain perception [28,29]. 

### 3.3. Modulation and Projection

Modulation, or integration, occurs when the stimulus reaches the dorsal horn of the spinal cord. This process attenuates the transmission in a phenomenon that can be produced at distinct levels. Transmission continues along the ascending (spinal-encephalic) pathways of the spinal cord to the neuronal relay centres that are involved in certain aspects of the experience of pain and stress, including the rostro-ventral medulla nucleus (pain, temperature information), bridge nucleus (pressure) and mesencephalic nucleus (proprioception). These superior centres are responsible for creating aspects of the pain experience through their action on a feedback circuit called the descending pain modulatory system [20]. During modulation, excitatory and inhibitory mechanisms alter the transmission of the nervous impulse [20]. Sensory information is then transported to the brain through projection neurons that begin in the laminae of the dorsal horn. Two supraspinal structures called the spinothalamic and spinoreticular tracts are of key importance in projection to higher brain regions [20]. 

### 3.4. Perception

The final stage is perception, which refers to the processing and integration of the information from several areas of the brain, including the cerebral cortex, to define the sensory characteristics of the painful stimulus: namely origin, localisation and type of stimulus [20]. It is important to consider that pain is multi-dimensional, with not only nociceptive and nocifensive (motor action) components, but cognitive and emotional components [30]. ‘Pain’ messages being relayed up the spinal cord are received at the thalamic nuclei. Pain information is then sent to cortical and subcortical regions, including the amygdala, hypothalamus, periaqueductal grey and areas of the cerebral cortex. The subjective experience or perception of pain is likely associated with activation of these cortical regions, but the experience can be modulated by these thalamic and limbic system connections [31]. For example, the amygdala has an important role in the emotional-affective component [30] but can also influence cognitive aspects such as decision-making in relation to pain [5,32,33,34]. 

The pain perception stage is of particular interest due to the effect that central sensitisation particularly has on modifying the response of the nervous fibres. This response is characterised by cascades of excitatory neurotransmitters such as glutamate, aspartate, catecholamines, prostaglandins, prostacyclins, bradykinin, substance P, interleukins and leukotrienes which are produced within the second order neurone. These substances diffuse back to the primary afferent fibre and generate hyperalgesic or even allodynic responses through actions at NMDA, AMPA and kainate receptors [5]. Hyperalgesia triggers stimulation of brain regions such as the cerebral cortex and reticular formation, to cause heightened pain perception [6,8,23,35]. 

A priority for mitigating pain around the time of slaughter is therefore to control the events and factors that lead to this ramping up of the pain response. The consequences of this are prolonged pain, and time to death, due to the perception of high-intensity harmful stimuli. The following sections specifically consider the impact of slaughter and associated events on the pain pathway.

## 4. Hyperalgesia and Events at Slaughter 

### 4.1. Hyperalgesia and Transport and Handling

During loading, transport and unloading, animals may be injured or exacerbate existing lesions resulting in inflammation [36]. Evidence for this as a potential concern in the context of slaughter comes from a number of studies. Herzberg et al., 2020 [37] reported elevated concentrations of cytokines including tumour necrosis factor (TNF-α), IL-1, IL-13, IFN-α and IFN-γ in Holstein-Friesian dairy cows with lameness compared to sound cows. Furthermore, it has been shown that the presence of previous pathologies or chronic injuries, such as metritis or pneumonia (associated with cytokine release), modifies the action potential of the nerve endings and triggers harmful responses of higher intensity [38]. However, in a recent study [39], the health status of 237 cull cows was evaluated at slaughter, along with specific slaughter-focussed welfare assessment indices. An indirect relationship between health status and carcass bruising was demonstrated; mammary issues and lameness predisposed to low body condition with the latter being a risk factor for bruising. Bruising might be assumed to be associated with inflammatory cytokine production and therefore lead to peripheral sensitisation, affecting behaviour at stunning. A potential outcome of this could be ineffective stun or need for second stun due to movement. However, haematoma number and severity was not associated statistically with welfare-specific variables recorded during the stunning process, such as requirement for a second shot, or increased time between stun and stick. Therefore, further large-scale epidemiological research is needed to elucidate the actual effects of inflammation on pain at slaughter.

The conditions under which animals are transported to the abattoir can also affect their behaviour often manifesting in dominant aggressive behaviours [40]. This predisposes to skin lesions such as haematomas and bruising. A study of water buffaloes transported under conditions of acute nociceptive stress found a total of 244 contusions or concussions, distributed in the following categories of bruises: 9.8% small, 59% small but deep, 19.3% medium, 6.1% medium but deep and 5.7% large. The areas of the body most often affected were the hind limbs, abdomen, shoulders, neck, back and perineum [41]. Similarly, a study by Ahsan et al. (2014) [42] found that 89% of cows and buffaloes presented at slaughter with lesions on the back, in the ventral area, at the base of the tail, and in the lumbar, thoracic and scapular regions. These were determined to be caused by other animals and/or contact with the transport vehicle and were prevalent when animals were forced to travel at densities much higher than recommended levels. These findings are of concern since such injuries will lead to nociceptor stimulation in the skin, and the underlying tissues depending on the depth of injury potentially leading to peripheral and central sensitisation, as described earlier. Risks of enhanced sensitisation are exacerbated by prolonged trip length due to the increased likelihood of animals being injured by others, by contact with the vehicle, or by movement.

**Figure 3:**
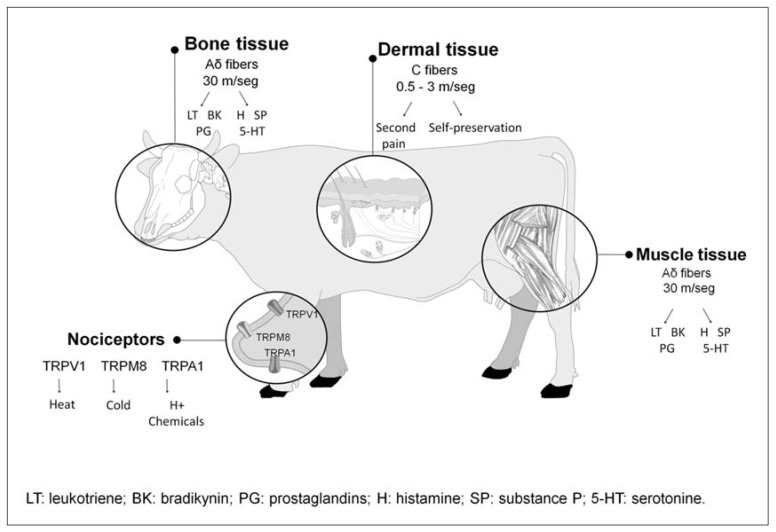
Schematic representation of the neurophysiological process of pain nociception in relation to the injured tissue and degree of trauma. Grade 1 trauma involves the skin, Grade 2 trauma has muscle involvement with the most severe (grade 3) trauma reflecting bone damage.

Another important consideration in relation to injury and sensitisation, as the central topic of this paper, is that location of injury and degree of trauma influences the sensitisation response (Figure 3). A grade 1 trauma which only damages the skin stimulates mechanoreceptors and thermoreceptors without producing a peripheral sensitisation response or hyperalgesia. However, in cases of grade 2 trauma where damage to muscle tissue occurs, polymodal receptors are stimulated in addition to nociceptors. This leads to a modified transduction and transmission process, whereby a mechanical stimulus causes a chemical response, mediated by chemoreceptors sensitive to the release of excitatory neurotransmitters [5,39,43]. More severe or grade 3 trauma, characterised by bone damage typically caused during transport or pre-slaughter handling, leads to both peripheral and central sensitisation of the nociceptive arch. 

Stress and blows might not end with transport. Animals may be struck by the guillotine door at the entrance to the stunning box and can be subject to poor handling practices and inadequate behaviour of some workers [44], including shouting, having their tails twisted to encourage movement, receiving blows to thoracic limbs and hindquarters [45], and excessive use of electric drivers [46]. Previous studies have identified that the most frequent animal behaviours in the stunning box are struggling (38.3%) and falling (9.5%) [47]. Since, these reactions can lead to pain an important recommendation is to perform desensitisation within the first 5 s after restraint is applied [47]. Animals that receive more positive human contact on farm during their early life have been shown to perform fewer resistance movements in the stunning box, as well as exhibiting greater forward movement [48]. In this regard, animal fear reactions to humans are a significant contributor to poor welfare at slaughter [49]. Another consideration in regard to early life experience is that experiences during development [25], such as chronic poor welfare or mistreatment, may lead to altered functioning of the HPA axis, affecting both its regulation and output. [25] This dysregulation leads to both central and peripheral sensitisation as described earlier. Therefore, in improving welfare at slaughter, consideration should not just focus on the abattoir environment but prior on-farm environment and the nature of human-animal interactions created there.

The OIE prohibits the use of electrical instruments to move small pigs, sheep and horses [48]. In cases where they are required, the OIE only allows battery-powered instruments with a maximum voltage of 30 V [50]. Drive use typically occurs when animals refuse to move even though there is space for them to do so, when they lie on the floor, obstruct the circulation of other animals, or when they fall during transport and must be made to stand up to prevent injuries caused by trampling [51]. Intense nociceptive stimulation may result from the application of electric prods on certain areas of the body, such as an animal’s face or genitals [47,52]. The occurrence of this event likely differs in frequency based on geographic region and local factors. Tissue damage generally correlates with time in contact with the drive, based on exposure time to electrical current. This contact time will influence the nature of the sensitisation processes occurring through the mechanisms discussed earlier, and dependent on location of injury. Electricity can also cause electrothermal wounds at the point of contact and in internal organs [53]. 

### 4.2. Hyperalgesia and Stunning

It has been suggested that following sticking in cattle, consciousness may last for 60 s or more [54]. During this period there is potential for animals to experience both pain and distress due to transection of a series of nociceptive fibers, that when activated generate a stream of sensory impulses that make pain perception inevitable [2]. In most jurisdictions, regulatory and guidance documents require that steps are put in place to mitigate the experience of pain associated with sticking. This is usually done by requiring pre-stick stunning [55,56,57]. 

The stunning process, or desensitisation, when performed correctly, renders animals unconsciousness until death supervenes. During this period of unconsciousness nociceptors continue to transduce harmful stimuli, but the brain can neither receive the stimulus nor perceive the pain generated because ascendent nervous transmission is inhibited [58,59]. An inadequately applied stunning method or rapid return of sensitivity can trigger a repetitive stimulation of the nociceptors due to cellular damage or extensive inflammation that lowers the threshold required for their activation. During this harmful phenomenon, poorly-stunned animals may develop primary hyperalgesia characterised by an exaggerated, prolonged response to a harmful stimulus that impacts the periphery. This is visualised clinically by an increase in the perception of, and sensitivity to, pain in the injured zone. Secondary hyperalgesia development may follow [22]. For this reason, is important to check for signs of recovery of consciousness in stunned animals and to perform a re-stun immediately when these are found. A significant number of cross-species studies have examined the causes and impact of inefficient stunning on animal welfare. It is beyond the scope of this review to examine all of this literature and the reader is referred to several excellent reviews on the topic [60,61,62]. Some pertinent points are presented here.

Gibson et al. (2015) [22] assessed the mechanical factors that impact stunning efficacy with a penetrating captive bolt gun using goat and cattle manikins. Kinetic energy values needed for effective stun are higher as animal size increases and ability to achieve this varies with make of captive bolt gun used. They also determined that repeated firing caused heating to 88.8ºC, which reduced stun effectiveness by decreasing the depth of penetration. This evidence highlights the need to assess the following aspects in consideration of stun efficacy: the amount of kinetic energy delivered to the animal’s head, depth of penetration, species and relevant anatomical considerations, and the time between stunning and sticking. 

**Figure 4:**
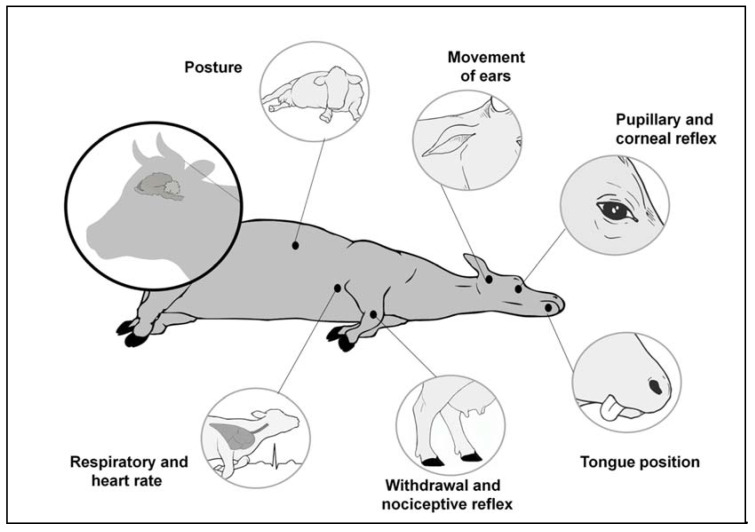
Indicators during slaughter caused by the absence, or poor application, of stunning. Visible indicators include vocalisations, movements of the ears, struggling, bristling, shaking, ocular and pupillary reflexes, the painful withdrawal stimulus, body posture, straightening reflexes, respiratory rhythm, the tongue position and the reflex reaction to a painful stimulus.

Gregory et al. (2007) [63] examined in greater detail the depth of penetration after captive bolt stunning. It was found that soft sound shots with 4.5-g cartridges were associated with shallow cerebral concussions that resulted in outward signs of inefficient stun such as failure to achieve physical collapse, presence of the corneal reflex, and post-collapse vocalising and nystagmus (Figure 4). Absence of tongue protrusion was determined to be an unreliable indicator of the depth of concussion. 

Animal species anatomy should be considered in relation to selected stunning technique, since certain methods may be superior. Collins et al. (2017) [64] compared the soft tissue and bone damage caused by applying a penetrating vs. a non-penetrating captive bolt gun by testing them on the cadavers of 12 crossbred female goats. Effectiveness was evaluated by magnetic resonance and computed tomography to examine the effects of the shots on the bony and soft tissues of the head. Both methods produced fractures in the occipital and interparietal regions, with structural damage to the soft tissues adjacent to the shot site being apparent. This damage was prevalent in the cerebellum, mesencephalon, myelencephalon, diencephalon and occipital lobes, and was accompanied by haemorrhaging in the underlying soft tissues. Penetration must reach the thalamic region to produce unconsciousness or insensibility to pain [65]. This may not be achievable using commonly used bovine captive bolt gun in buffalo. Schwenk et al. (2016) [65] evaluated brain injury caused by the captive bolt method in water buffaloes, finding that the depth of penetration, measured by magnetic resonance, was not sufficient to reach the thalamus. Furthermore, sex may influence risk of ineffective stun with an increased risk of failing to induce motor paralysis being found in bulls, regardless of the actual level of deviation of bolt entry from the ideal position [66]. This finding is postulated to arise due to the relatively thicker bone mass on the forehead in bulls, which provides increased resistance to the kinetic energy delivered by the bolt [66]. Other studies have similarly reported an increased chance of ineffective stun in bulls [63,67]. However, in contrast Gouveia et al. 2009 reported the opposite with a higher proportion of unsuccessful stuns in females [68]. These findings may however have been biased due to differences in age distribution of animals in their examined population, with a relatively greater proportion of older females. 

Electrical stunning is designed to disrupt the normal rhythmic electrical activity in the brain produced by cerebral neurons [8]. Correct application leads to synchronous de- or hyperpolarization of neurons leading to an epileptiform seizure. This seizure activity originates in the interconnections between the thalamus and cortex, and the brainstem, and then extends out to other nearby structures [69]. Reversibility and duration of the seizure is dependent on the current passed and the brain areas affected. Akin to mechanical stunning, an advantage of electrical stunning is the possibility that unconsciousness might be achieved almost instantaneously. However, failures arise due to insufficient electric current reaching the brain as a result of inadequate equipment maintenance or poor electrode placement [70]. In the case of improper stunning, events related to sensitisation can occur in the absence of unconsciousness. For example, adrenaline is released in large quantities contributing to a modified pain pathway [71]. Furthermore, head–only or head-back stunning may, dependent on species, cause intense muscle contractions which can lead to bone fractures, tearing and bruising of the muscle tissues, as well as blood pressure increases which serves to increase bruising [72,73]. These events trigger peripheral sensitisation processes and subsequent secondary hyperalgesia, heightening pain responses until death supervenes or a second stun is received. 

### 4.3. Hyperalgesia and its Relationship with Slaughter Performed without Prior Stunning

Religious slaughter requires a single cut to the neck severing the jugular veins and carotid arteries bilaterally, to exsanguinate the animal and induce loss of consciousness due to cerebral ischemia. This is done in accordance with rules laid down in the holy texts (the Koran or the Torah) [15]. Religious slaughter without stunning is allowed in many countries, but this may not be the only reason for slaughter without stunning. For example, emergency or backyard slaughter may occur without pre-stun [74].

Issues of concern specific to slaughter without stun are: 1) the cutting of the neck, blood vessels and surrounding tissues, without pre-stun and 2) the immediate post-cutting period [75]. Pain and distress arise due to the incision itself, which activates various nociceptors, but also due to sustained activation of the SAM system due to hypotension caused by bleeding. This causes tachycardia, tachypnea, hyperthermia and a redistribution of the blood [2,23].

Imlan et al. (2020) [2] evaluated the effects of the knife edge on biochemical parameters, plasma catecholamines and electroencephalographic responses (EEG) of Brahman crossbreed steers after neck-cutting. Their procedure entailed cutting the carotid supply routes, jugular veins, trachea and throat during Halal religious slaughter, where prior stunning was not used. A significant increase in post-slaughter adrenaline, glucose, kinase creatinine and lactate dehydrogenase levels occurred when a commercial knife was used compared to a knife sharpened and then tested by a method designed to ensure optimum sharpness. The EEGs showed significant increases in median frequency and total power when the commercial knife was used, indicating that animals experienced greater pain and stress under that condition. This confirms that neck-cutting leads to the harmful stimuli being perceived integrally in both the cerebral cortex and reticular formation [23]. In goats, electroencephalographic changes similarly demonstrate that sticking causes noxious stimulation, regardless of whether animals are conscious or unconscious at the time of stick [76]. 

Effective stunning, with quick disgorgement prevents pain perception occurring in the higher brain regions, evidenced experimentally by lack of EEG response [77]. Stunning may also prevent elevations in stress hormones [78], which can contribute to sensitisation via various mechanisms as described earlier. Bozzo et al. (2018) [79] compared the plasma cortisol and catecholamine concentrations of 60 Charolais male beef cattle slaughtered following either traditional method (with stunning prior to neck-cutting) or the Kosher method. They evaluated three stages: on- farm, post-transport and exsanguination, with the finding that whilst on farm and post-transport cortisol and catecholamine levels were low, during exsanguination the levels of both substances were 50% higher in the animals slaughtered by the Kosher method than in those stunned prior to slaughter. Alternately, no significant differences in catecholamines were found between goats either slaughtered conscious or after minimal anaesthesia [76]. Care also has to be taken in interpreting such findings since these changes may not necessarily equate with pain [78]. Pain represents a source of stress which will activate the physiological stress systems. However, stress at slaughter does not only arise from a physical cause, such as a lesion creating pain [8]. Stress may also arise from fear reactions created by mixing with unfamiliar animals and human contact, as well as those created by the unknown environment and events of slaughter [80]. 

During sticking the animal experiences prolonged nociceptive stress associated with the time taken to onset of unconsciousness. Moreover, blood may also be aspirated into the airways causing respiratory distress [3,10]. Gregory et al. (2009) [81] examined the airways of cattle slaughtered by religious slaughter and slaughter with prior stun using penetrating captive bolt. The animals which were not stunned continued to breathe during the first part of the bleeding process, in contrast to the ones that were stunned which decreased their rate and depth of breathing during exsanguination. In that study, 19% of the animals slaughtered by the Shechita method, 58% of those slaughtered by the Halal technique and 21% of those that were stunned had accumulations of blood in the trachea. In addition, 36%, 69% and 31%, respectively, presented blood in the bronchial tract, and 10%, 19% and 0% respectively, had fresh blood in the trachea. On the basis of these findings, the researchers concluded that slaughter without stunning caused respiratory distress [81]. The entrance of liquid –in this case blood– into the bronchial tract and alveolar space triggers a reaction to release pro-inflammatory cytokines, TNF-α and interleukins such as IL-1 and IL-10. This inflammatory soup may then contribute to sensitisation, although time course of this may be prolonged and therefore of less relevance in the context of effects at sticking. The literature is controversial on effects of blood aspiration and slaughter distress. While some authors claim that aspiring blood through the upper airways and lungs during slaughter causes intense suffering if the animal has not been stunned, others advance that no suffering occurs because the afferent signals activated by the presence of irritants in the lungs are regulated by neurons of the vagus nerve which have been severed by the cut [82]. However, in conscious animals with intact vagus nerves, the presence of liquid in the respiratory tract causes irritation of nociceptors in the airways, especially those of the glottis, larynx and trachea, to provoke coughing or activate reflexes to expel the foreign substance [83]. Therefore, risk of distress is increased if the vagus nerves are not completely transected. 

Level of the transection may play a role in distress caused by fluid entry to the respiratory tract. The laryngeal reflex is regulated through neuronal connections in the C2 cervical vertebra, but in the Halal and Shechita methods the neck is cut between C3 and C5 [83], so this reflex continues to be present and active in animals that are conscious due to lack of stunning. Cut level is then potentially relevant to suffering in these animals and presents an opportunity for method refinement to improve welfare. 

The time to collapse after slaughter is another consideration relevant in comparisons of pre-stun and non-stun methods. Gregory et al. (2010) [3] reported that 14% of non-stunned cattle collapsed but stood up again before collapsing definitively, whilst 8% took 60–75 s to collapse. These results are suggestive that slaughtering animals without stunning increases the risk for a prolonged period of pain and distress. Zulkifli et al. (2014) performed a comparative analysis of the effects of penetrative stunning, non-penetrative stunning and post-slaughter stunning on biochemistry and the EEG in cattle [84]. Plasma noradrenaline concentrations increased in all animals. In addition, post-slaughter plasma ACTH concentrations in the animals stunned by the captive bolt method, followed by Halal slaughter were significantly higher than in the other study groups, likely indicating a physiological response to stress. Based on the EEG results obtained, penetrative stunning maximised the possibility of desensitisation post-stunning, while the animals stunned after the Halal cut showed t increases in encephalographic activity consistent with the presence of harmful post-slaughter stimuli associated with sectioning and lesioning of tissues. The researchers concluded that penetrative stunning was the most reliable method for ensuring insensitivity by minimising transduction, transmission, projection and perception of pain.

## 5. Conclusions

The use of poor transport and handling methods prior to slaughter, as well as the application of unsuitable stunning methods, may result in processes of sensitisation –central or peripheral– which intensify pain perception. In addition, animals destined for slaughter may be suffering from previous pathologies, the sequelae of which could contribute to sensitisation. Even where suitable stunning methods are utilised, deficiencies in handling prior to application of an effective stun, poor equipment maintenance and/or inadequate personnel training could also result in nociceptor stimulation, and trigger processes of peripheral sensitisation and pain perception. Further dedicated study is needed to confirm the hypothesis of a link between events at slaughter and processes of hypersensitisation. 

## Data Availability

No data collected.

## Abbreviations/Nomenclature

5-HT: serotonin; AMPAR: α-amino-3-hydroxy-5-methyl-4-isoxazolepropionic acid receptor; AP: action potential; ASIC; acid-sensing ion channels; ATP: adenosine triphosphate; BDNF: brain-derived neurotrophic factor; BK: bradykinin; CGRP: calcitonin gen related peptide; GLU: glutamate; GPCR: G-protein-coupled receptors; HI: histamine; IFN: Interferon; IL: interleukin; NGF: nerve growth factor; NK1R: neurokinin-1 receptor; NMDAR: N-Methyl-D-aspartate receptor; NO: nitric oxide; PG: prostaglandin; SP: substance P; TNFα: tumour necrosis factor; TrkA: tropomyosin receptor kinase A; TRPV: transient receptor potential cation channel.
